# Dexamethasone Iontophoresis for Musculoskeletal Tendinopathies: A Case Series of Three Patients

**DOI:** 10.7759/cureus.107408

**Published:** 2026-04-20

**Authors:** Lily Kharlamb, Rachel B Ikhilov, Yevgenia J Gitlin-Nitti

**Affiliations:** 1 Biochemistry, City University of New York Graduate Center, New York, USA; 2 Pharmacy, Long Island University, Brooklyn, USA; 3 Physical Medicine and Rehabilitation, Miami Veterans Affairs Healthcare System, Key West, USA

**Keywords:** achilles tendinopathy, de quervain's tenosynovitis, dexamethasone, iontophoresis, lateral epicondylitis, physical therapy, tendinopathy, transdermal drug delivery

## Abstract

Iontophoresis is a non-invasive transdermal drug delivery technique that uses low-voltage electric current to deliver ionized medications into soft tissues. Despite guideline recommendations supporting dexamethasone iontophoresis for tendinopathies, the existing evidence consists primarily of condition-specific randomized controlled trials and single-site retrospective studies, with no published case series illustrating the real-world application of dexamethasone iontophoresis across multiple tendinopathy types within a single report. This case series describes the outcomes of dexamethasone iontophoresis as part of multimodal physical therapy for three distinct tendinopathies.

This retrospective case series included three patients treated at a Veterans Affairs physical therapy clinic with dexamethasone sodium phosphate (4 mg/mL) iontophoresis (40 mA-min per session) using the ActivaDose II system. While the small sample size limits generalizability, this series is intended to illustrate the real-world clinical application of dexamethasone iontophoresis across multiple tendinopathy types rather than to establish efficacy. Concurrent interventions included therapeutic exercise, patient education, and orthotic management. Outcomes assessed included pain (0-10 numeric scale), range of motion, grip strength, and Upper Extremity Functional Index (UEFI) scores.

Case 1 (de Quervain's tenosynovitis) is a 47-year-old man with persistent, functionally limiting wrist pain who completed seven sessions over 24 days. Pain decreased from 3-4/10 to 1/10, grip strength improved from 60 to 75 lbs (equalizing with the uninvolved side), and UEFI improved by 8 points (exceeding the MCID). Case 2 (fluoroquinolone-associated Achilles tendinopathy) is a 56-year-old woman who completed six sessions over nine weeks. Pain decreased from 8/10 to 2-3/10, and ankle dorsiflexion improved from 7° to 16° (left) and 10° to 20° (right), surpassing the normative threshold of 10°. Case 3 (lateral epicondylitis) is a 62-year-old man with acute-onset symptoms who completed 12 sessions over six weeks. Pain decreased from 8/10 to 2-3/10, grip strength improved from 22 to 110 lbs (exceeding the uninvolved side of 80 lbs), and UEFI improved by 46 points (exceeding the MCID). No adverse effects were reported.

Dexamethasone iontophoresis, as part of comprehensive physical therapy, was associated with clinically meaningful improvements in pain, strength, and function across three tendinopathy types. These findings suggest that dexamethasone iontophoresis may serve as a useful non-invasive adjunct in the multimodal management of musculoskeletal tendinopathies, though prospective controlled studies are needed to confirm its independent contribution.

## Introduction

Iontophoresis is a non-invasive transdermal drug delivery technique that utilizes a low-voltage electric current to facilitate the movement of ionized medications across the skin barrier into soft tissues [1-3]. This method involves electromigration (movement of charged drug molecules in an electric field) and electroosmosis (bulk fluid flow carrying drug molecules), which together facilitate deeper tissue penetration than passive diffusion alone, and is increasingly used in the treatment of musculoskeletal disorders to target pain and inflammation while minimizing systemic exposure [1,3-5]. Gurney and Wascher demonstrated that iontophoresis achieves therapeutically relevant dexamethasone concentrations in human tendon tissue (2.9 vs. 0.2 ng/g with sham; p=0.02), providing a pharmacokinetic rationale for its clinical application in tendinopathies [4].

A range of pharmacological agents can be delivered via iontophoresis, including corticosteroids (e.g., dexamethasone), non-steroidal anti-inflammatory drugs (NSAIDs), and local anesthetics, with drug selection and electrode polarity determined by the compound's ionic charge [3,5,6]. The amount of drug delivered is substantially greater than with conventional topical application, and the technique has been used in various specialties, including physical therapy, dermatology, and dentistry [1,3,5,6]. 

In musculoskeletal practice, iontophoresis has demonstrated efficacy in lateral epicondylitis (tennis elbow), Achilles tendinitis, plantar fasciitis, and subacromial impingement syndrome [7,8-19]. In Achilles tendinitis, clinical evidence supports the efficacy of iontophoresis with dexamethasone for tendinopathies, with randomized controlled trials demonstrating significant improvements in pain, strength, and function [8-10,15,18]. These findings suggest that iontophoresis may serve as a practical adjunct in physical therapy for select musculoskeletal indications, though further controlled studies are needed to confirm its independent contribution.

Despite clinical practice guideline recommendations such as the American Physical Therapy Association's Achilles tendinopathy clinical practice guideline published in the Journal of Orthopaedic and Sports Physical Therapy, published case series demonstrating the real-world application of iontophoresis with dexamethasone across multiple tendinopathy types remain limited [8].

The choice of medication for iontophoresis depends on the clinical indication and available evidence. Among medications used in iontophoresis therapy for musculoskeletal conditions, dexamethasone has the strongest clinical evidence base. Diclofenac and ketoprofen have both demonstrated anti-inflammatory efficacy via iontophoretic delivery, with diclofenac having more published human clinical data, though no head-to-head trials have established comparative superiority between the two NSAIDs. Lidocaine may be used as an adjunctive agent for analgesia. Evidence for other agents in musculoskeletal indications remains limited. Lidocaine can be used as a secondary adjunct therapy. Limited clinical evidence exists to support the use of other pharmacological agents for musculoskeletal iontophoresis at this time [4,9,10,12-15,17,19]. 

Dexamethasone is the most frequently and clinically supported drug used in iontophoresis for musculoskeletal conditions. Clinical practice guidelines published in the Journal of Orthopaedic and Sports Physical Therapy recommend iontophoresis with dexamethasone for conditions including lateral epicondylitis, acute and insertional Achilles tendinopathy, subacromial impingement syndrome, and plantar fasciitis [8]. Patients demonstrated reductions in pain and improved function using iontophoresis with dexamethasone [4,8,10,13-19]. In a landmark randomized, double-blind, placebo-controlled trial of 199 patients with lateral epicondylitis, Nirschl et al. demonstrated that dexamethasone iontophoresis (six treatments of 40 mA-minutes) produced significantly greater pain reduction and functional improvement compared with placebo [17]. The therapeutic doses used in clinical trials range from 1 to 4 mg/mL treatment over 10- to 15-minute sessions at 0.1-0.2 mA/cm [7,10,13-15,17]. 

NSAIDs, including diclofenac and ketoprofen, have been used in iontophoresis to reduce local inflammation and pain while minimizing gastrointestinal and other adverse effects associated with oral administration. Diclofenac has been evaluated in several human clinical studies, while ketoprofen has demonstrated enhanced tissue penetration in the preclinical models of osteoarthritis and pain. No head-to-head comparative trials between iontophoretically delivered NSAIDs have been conducted, highlighting the need for future studies to establish relative efficacy and optimal agent selection [5,6,9,12].

Lidocaine, a local anesthetic, can be used in iontophoresis. It has been found to be effective as an adjunctive therapy commonly used with dexamethasone. Randomized controlled trials show the successful absorption of 2% lidocaine delivered via iontophoresis, and effectiveness has been specifically noted in lateral epicondylitis [19].

## Case presentation

This case series describes three patients with distinct tendinopathies treated with iontophoresis with dexamethasone as part of comprehensive physical therapy management. Although a case series of this size cannot establish efficacy, it can provide clinically relevant observations to inform practice and guide future prospective research. This retrospective case series was reviewed by the Institutional Research and Development (R&D) Committee at the Miami Veterans Affairs Healthcare System, which determined that institutional review board (IRB) approval was not required as the study involved review of de-identified clinical data and met the criteria for exemption under institutional policy. Informed consent was waived by the R&D Committee due to the retrospective, de-identified nature of the data. Patient identifiers have been removed to protect privacy.

Diagnoses were established clinically based on history and physical examination findings. Imaging studies were not obtained as they are not routinely required for the clinical diagnosis of these tendinopathies and were not indicated based on the patients' presentations. All patients received dexamethasone sodium phosphate (4 mg/mL) delivered via the ActivaDose II iontophoresis system. The active electrode (cathode) was saturated with approximately 1-2 mL of dexamethasone sodium phosphate solution and placed directly over the site of maximal tenderness at the affected tendon. The dispersive electrode (anode) was placed approximately 4-6 inches proximal or distal to the active electrode. Each treatment session delivered a total dose of 40 mA-min at a current intensity of 2.0-4.0 mA over approximately 10-20 minutes, consistent with published clinical protocols. Treatment parameters were standardized across all three cases.

Each patient also received concurrent therapeutic interventions including exercise prescription, patient education, and orthotic management as clinically indicated. As this case series was designed to describe real-world clinical application rather than to isolate the independent effect of iontophoresis, the contribution of each individual intervention cannot be determined. Outcome measures collected at initial evaluation and discharge included pain ratings (0-10 numeric scale), range of motion (goniometry), strength testing (dynamometry and manual muscle testing), and functional assessments using the Upper Extremity Functional Index (UEFI) for upper extremity cases. For the purpose of this publication, outcome measures were collected at initial evaluation and discharge. Intermediate progress data were not systematically recorded as part of routine clinical documentation and therefore are not reported.

Case 1: de Quervain's tenosynovitis

A 47-year-old man with an occupation involving prolonged computer use presented with gradual-onset right wrist pain over several months, localized to the radial aspect of the wrist and thumb. He reported pain rated 3-4/10 at worst with computer mouse use and video gaming. The patient had not received prior treatment for this condition. Symptom duration was approximately three months. Examination revealed a positive Finkelstein test, tenderness over the abductor pollicis longus and extensor pollicis brevis tendons, limited combined thumb adduction with ulnar deviation, and reduced grip strength (60 lbs right vs. 70 lbs left). Normal grip strength for men aged 45-49 is approximately 100-110 lbs for the dominant hand. UEFI score was 69/80. Carpal tunnel syndrome and intersection syndrome were considered but excluded based on the absence of numbness/tingling and the localized tenderness pattern. de Quervain's tenosynovitis was diagnosed clinically based on radial-sided wrist pain, tenderness over the first dorsal compartment, and a positive Finkelstein test, consistent with established clinical diagnostic criteria.

The patient received seven physical therapy sessions over 24 days including iontophoresis with dexamethasone, therapeutic exercise, ergonomic education, and thumb spica bracing. At discharge, pain decreased from 3-4/10 to 1/10 (67-75% reduction), grip strength improved from 60 to 75 lbs (25% improvement), and UEFI score increased to 77/80. Range of motion improved across all measured planes and was symmetrical to the uninvolved side, with the exception of a minimal residual limitation in combined thumb adduction with ulnar deviation. Specific goniometric values for this composite movement were not recorded, as clinical assessment focused on functional symmetry and pain-free motion.

Case 2: Achilles tendinopathy

A 56-year-old woman developed bilateral Achilles pain approximately six months after taking levofloxacin, with the left side more symptomatic. Fluoroquinolone-associated tendinopathy is a well-documented adverse effect of levofloxacin. No prior physical therapy or corticosteroid injections had been administered for the Achilles symptoms. She reported pain rated 8/10 at worst after prolonged sitting, with morning stiffness and first-step pain. Examination showed bilateral Achilles tenderness (left > right), limited ankle dorsiflexion (7° left, 10° right; normal >10°), and pain aggravated by wearing flip-flops but relieved with supportive footwear. Achilles rupture and retrocalcaneal bursitis were excluded based on an intact Thompson test, absence of posterior heel swelling, and localized tenderness pattern. Achilles tendinopathy was diagnosed based on localized Achilles tenderness, morning stiffness, and pain with activity, associated with fluoroquinolone use.

The patient completed six physical therapy sessions over nine weeks including iontophoresis with dexamethasone, progressive stretching and eccentric strengthening exercises, and night splinting. At discharge, pain decreased from 8/10 to 2-3/10 (63-75% reduction), ankle dorsiflexion improved from 7° to 16° on the left (129% improvement) and from 10° to 20° on the right (100% improvement), and tenderness reduced to mild on the left Achilles only.

Case 3: lateral epicondylitis

A 62-year-old man developed acute right lateral epicondylitis after two days of intensive window installation work. He reported severe pain (8/10 at worst) with morning stiffness, difficulty extending the elbow, and pain with lifting a coffee cup. The patient had not received prior treatment. Symptom duration was approximately two weeks at initial evaluation. Examination revealed significant lateral epicondyle tenderness, markedly reduced grip strength (22 lbs right vs. 80 lbs left). Normal grip strength for men aged 60-64 is approximately 80-90 lbs. The patient had limited wrist extension (54° vs. 68° left). Normal wrist extension ranges from 60° to 70°, with a UEFI score of 31/80. Radial tunnel syndrome was considered but excluded based on the location of maximal tenderness and absence of pain with resisted supination. Lateral epicondylitis was diagnosed based on lateral epicondyle tenderness, pain with resisted wrist extension, and acute onset following repetitive manual activity.

The patient underwent 12 physical therapy sessions over six weeks including iontophoresis with dexamethasone, progressive stretching and eccentric strengthening, and orthotic management (tennis elbow strap, elbow extension sleeve, and Pil-O-Splint for nighttime use). At discharge, pain decreased from 8/10 to 2-3/10 (63-75% reduction), grip strength improved from 22 to 110 lbs (400% improvement), exceeding the uninvolved side, wrist extension normalized, and UEFI score increased to 77/80.

A summary of patient demographics, interventions, and outcomes across all three cases is presented in Table 1.

**Table 1:** Summary of patient demographics, interventions, and clinical outcomes DF: dorsiflexion; WE: wrist extension; UEFI: Upper Extremity Functional Index; ROM: range of motion; N/A: not applicable (lower extremity case)

Variable	Case 1: de Quervain's tenosynovitis	Case 2: Achilles tendinopathy	Case 3: lateral epicondylitis
Age/sex	47/male	56/female	62/male
Symptom duration	~3 months	~6 months	~2 weeks
Baseline pain (0-10)	3-4 out of 10	8 out of 10	8 out of 10
Discharge pain (0-10)	1 out of 10	2-3 out of 10	2-3 out of 10
% change in pain	67-75% reduction	63-75% reduction	63-75% reduction
Baseline grip strength	60 lbs (vs. 70 lbs uninvolved)	N/A	22 lbs (vs. 80 lbs uninvolved)
Discharge grip strength	75 lbs bilateral	N/A	110 lbs (vs. 80 lbs uninvolved)
% change in grip strength	25% improvement	N/A	400% improvement
Baseline UEFI	69/80	N/A	31/80
Discharge UEFI	77/80 (+8 points)	N/A	77/80 (+46 points)
Baseline ROM deficit	Limited thumb adduction/ulnar deviation	DF: 7° left and 10° right	WE: 54° (vs. 68° uninvolved)
Discharge ROM	Minimal residual limitation	DF: 16° left and 20° right	WE: 68° bilateral
Number of sessions	7	6	12
Concurrent Interventions	Exercise, ergonomic education, thumb spica brace	Stretching, eccentric strengthening, night splint	Stretching, eccentric strengthening, orthotic management

## Discussion

This case series demonstrates successful outcomes using iontophoresis with dexamethasone as part of multimodal physical therapy for three distinct tendinopathies: de Quervain's tenosynovitis, Achilles tendinopathy, and lateral epicondylitis. UEFI improvements of 8 points in Case 1 and 46 points in Case 3 both exceed the established minimum clinically important difference (MCID) of 6-11 points, as determined by anchor-based methods in a prospective cohort of 1,708 patients with musculoskeletal disorders, indicating clinically meaningful improvement. The combination of iontophoresis with exercise, education, and orthotics reflects real-world multimodal physical therapy practice, though the individual contribution of each intervention cannot be determined from this case series. All patients achieved clinically meaningful improvements in pain, strength, range of motion, and function. No adverse effects, including skin irritation, burns, or allergic reactions, were reported during treatment sessions or at the time of discharge. Safety was assessed by clinician inspection of the electrode application site before and after each session and by patient self-report throughout the treatment course. These outcomes align with published evidence supporting iontophoresis with dexamethasone for tendinopathies. Nirschl et al. demonstrated significant pain reduction and functional improvement with dexamethasone iontophoresis compared with placebo in 199 patients with lateral epicondylitis, and Neeter et al. reported improved pain and function in acute Achilles tendon pain treated with dexamethasone iontophoresis. The magnitude of pain reduction observed in our Cases 2 and 3 (5-6 points on a 0-10 scale) is consistent with the treatment effects reported in these controlled trials.

Limitations of this case series include the lack of a control group, small sample size, and inability to isolate the specific contribution of iontophoresis from other interventions. The variable treatment durations and follow-up periods also limit direct comparison between cases. Additionally, long-term outcomes beyond discharge were not captured.

## Conclusions

This preliminary case series suggests that iontophoresis with dexamethasone may be a beneficial component of comprehensive physical therapy management for musculoskeletal tendinopathies affecting different anatomical regions. All three patients demonstrated improvements in pain, strength, range of motion, and functional outcomes; however, these findings are based on a small, uncontrolled case series and cannot establish causality due to concurrent interventions. The non-invasive nature of iontophoresis, combined with its favorable safety profile observed in this series, suggests it may be a useful adjunct for patients with acute and subacute tendinopathies, though its independent contribution remains to be established. Future randomized controlled trials with larger sample sizes, standardized dosing and electrode placement protocols, and follow-up periods extending beyond discharge are needed to establish optimal treatment parameters, evaluate long-term durability of outcomes, and identify patient characteristics associated with the best response to dexamethasone iontophoresis.
